# Old Age and the Thyroid Gland

**Published:** 1919-10-25

**Authors:** 


### OLD AGE AND THE THYROID GLAND.

### A Criticism.

It is certain that to bring vital matters to the notice of the average man, reference to them should occur in the newspaper which he habitually studies each day, but at the same time it cannot be urged too strongly that his taste for medical writings, his interest in his own health, should be stimulated by methods other than the publication of sensational medical "copy." As in the instance referred to later, it is most frequently the foreign correspondents who have made use of dramatic announcements with a medical bearing, described marvellous new operations, and brought to light supposed extraordinary advances which will benefit the sick man, but even the most casual observer must have noticed that with remarkable regularity, nothing more is heard of these great happenings. Apparently it is only necessary that some doctor of greater or less distinction should, in the true interest of research, publish an outline of some work performed, some plans made, which he hopes will bear fruit in the future, and journalistic enterprise is ever in some form or other ready to complete the picture. The tendency to publish sensational medicine is by no means general in our Press; it has, in fact, greatly diminished of late, but these paragraphs and articles do still appear.

Let us take, for example, the latest instance of exaggerated reporting. In substance the public are told that by grafting certain gland tissues into the human body the surgeon will be able to re-endow the aged person with the long-lost physical abilities of youth, and in this connection the thyroid or para-thyroid glands are actually mentioned.

### The Technique of Grafting.

Now there is more than one point which is directly misleading in the developments implied. A contemporary, in criticism, aptly compares the imagined process to the grafting commonly performed upon fruit-trees which are past their prime. Here the vitality of the old trunk is such that it may become the foster-parent of vigorous young shoots of entirely different parentage, and the resulting whole is certainly remarkably like a complete rejuvenation. But the human body is not like an apple-tree, as far, at least, as the process of grafting is concerned. True it is that like tissue may be grafted to like tissue in a certain number of the simpler structures of the body—namely, bone to bone, skin to skin, cartilage to cartilage—but we have always experienced that more complicated organs, particularly glandular organs, will not allow of this process. Also that tissue cannot be successfully implanted in heterogeneous surroundings, and a piece of gland thus grafted in the body sooner rather than later fibroses or is absorbed. We are inclined to doubt, until much more definite evidence comes to hand, the accuracy of this report of gland grafting.

Next to return to the thyroid in particular. This structure is now accepted as being one of the most important and most interesting of the ductless or endocrine glands. It is one of those which apparently add to the circulating blood a substance the concentration and amount of which are of vital importance to the maintenance of perfectly balanced health. The exact details of this action, which physiologists are still in process of elucidating, need not concern us here. The point is that a pathogenic condition is produced by either an excess or a deficiency of this internal secretion, and except in marked and typical instances, the condition is by no means easily diagnosed clinically. A short time ago we gave a review of the multitude of small troubles which may arise from "minor thyroid deficiency," and it is indeed marvellous to observe the speed at which such cases recover under judicious administration of thyroid extract. On the other hand, there is hyper-thyroidism, a condition perhaps more marked, more readily recognised, and certainly more rapidly fatal to good health. Thus it is obvious that addition of extra thyroid secretion to the system must be under expert control and able to be stopped at will. Even were it possible to graft successfully portions of the gland into the body, it appears to us most unlikely that this very necessary control could be maintained, and it is probable that there is at present only one satisfactory way in which a deficient thyroid can be safely assisted, and that is by the therapeutic method now in use.

### The Process of Old Age.

Also, if we take the main journalistic point, old age and its loss of bodily vigour, it is more than doubtful whether the changes seen in this phase of life can be anything like generally attributed to thyroid deficiency. A thousand factors may contribute to the determination of rate of advance of "old age." For the thyroid, however, it may be said that there is evidence that upon the functional activity of this gland depends a great deal of the necessary destruction, and elimination from possibility of harmful activities, of many by-products of the complicated processes by which our bodies assimilate food. More technically it is a safeguard against some forms of auto-intoxication. Admittedly, if this auto-intoxication be prevented *from the outset*, the wear and tear on the tissues, the process of old age will be delayed, but in a person already old, all the changes which may or may not have arisen from thyroid deficiency have become established—fibroses, arterio-sclerosis, degenerations, and the rest—and at this stage no amount of extra thyroid principle will undo the harm that is already done. Thyroid therapy may improve the health at the time, and in expert hands the rapidity of further ageing processes may be appreciably retarded. The elderly patient may feel considerably better than he did, but he has not regained his youth.

